# Esophageal Perforation and Pneumomediastinum Due to Delayed Diagnosis of Esophageal Lichen Planus

**DOI:** 10.7759/cureus.35453

**Published:** 2023-02-25

**Authors:** Bhavik Hirapara, Daniel Witheiler, Paul R Tarnasky, Miguel Villamil

**Affiliations:** 1 Internal Medicine, Methodist Health System, Dallas, USA; 2 Dermatology, Methodist Health System, Dallas, USA; 3 Gastroenterology, Methodist Health System, Dallas, USA

**Keywords:** dermatologic manifestations, esophageal stricture, pneumomediastinum, esophageal perforation, esophageal lichen planus

## Abstract

Esophageal involvement of lichen planus is an under-reported and under-diagnosed manifestation that should prompt immediate treatment given its high rate of complications. We highlight a rare case of a 62-year-old Caucasian woman with history of known oral lichen planus and esophageal strictures presumed to be secondary to gastroesophageal reflux disease, that presented with esophageal food impaction resulting in perforation and subsequent pneumomediastinum after esophagogastroduodenoscopy (EGD). Further workup, including a repeat EGD, revealed that the esophageal strictures were rather a complication of lichen planus. The patient was started on oral, topical steroids and underwent serial esophageal dilations with improvement. Esophageal lichen planus should be high on the differential, especially in patients with involvement of other mucous membranes and strictures refractory to therapy. Complications such as recurrent esophageal strictures and perforation may be preventable with early diagnosis and adequate treatment.

## Introduction

Lichen planus is a dermatologic chronic inflammatory and immune-mediated disorder that affects the skin, hair, nails, and mucous membranes [1]. Skin lesions are characterized by shiny, violaceous, flat papules in middle-aged adults that typically remit and recur [1,2]. Mucous membrane lesions occur in approximately 30-70% of cases, with or without skin lesions [3]. They appear as white plaques, erosions, and ulcerations that involve the genital area, gastrointestinal tract, and conjunctival mucosa [1-3].

An under-reported manifestation of the disease is esophageal involvement, with less than a few hundred reported cases [4]. Typical presenting symptoms of esophageal lichen planus (ELP) are dysphagia and odynophagia. One or more esophageal strictures are often seen in esophagogastroduodenoscopy (EGD), particularly in the mid and proximal esophagus [5]. Most patients with ELP have concomitant oral disease with visible plaques or ulcerations on the tongue or buccal mucosa [4]. Diagnosis is often clinical, but biopsies are frequently done for confirmation and to exclude pre-malignant cells. Treatments vary from topical to systemic steroids for both oral and ELP, and strictures are often dilated for patients with dysphagia. Given the rarity of ELP, esophageal strictures are often misdiagnosed as secondary to other disease processes, such as gastroesophageal reflux disease (GERD), which leads to delays in treatment.

ELP is under-reported and under-diagnosed. There are even fewer reported cases of esophageal perforation in the setting of ELP [6]. We present a rare case of esophageal perforation after endoscopy in the setting of a stricture and food bolus impaction due to delayed diagnosis of ELP.

## Case presentation

A 62-year-old Caucasian female presented to an outside hospital with esophageal food impaction and inability to manage secretions. She had a past medical history of esophageal strictures for over 10 years requiring yearly esophageal dilations, GERD, oral lichen planus managed with dietary modifications and intermittent use of topical steroids, hypertension, depression, anxiety, and arthritis.

She was intubated and underwent an urgent EGD that was unsuccessful in removing the food impaction due to the inability to pass the scope through a stricture found above the foreign body. A mucosal rent was noted without evident perforation. Imaging demonstrated extensive pneumomediastinum, interstitial emphysema in the stomach, and free air along the lesser curvature of the stomach likely secondary to esophageal perforation.

The patient was medically managed with broad-spectrum intravenous antibiotics, extubated to nasal cannula, and transferred to our facility for further evaluation and management. Upon awakening, the patient felt that the food bolus had passed and no longer had difficulty managing secretions. In addition, serial imaging demonstrated improvement in pneumomediastinum.

**Figure 1:**
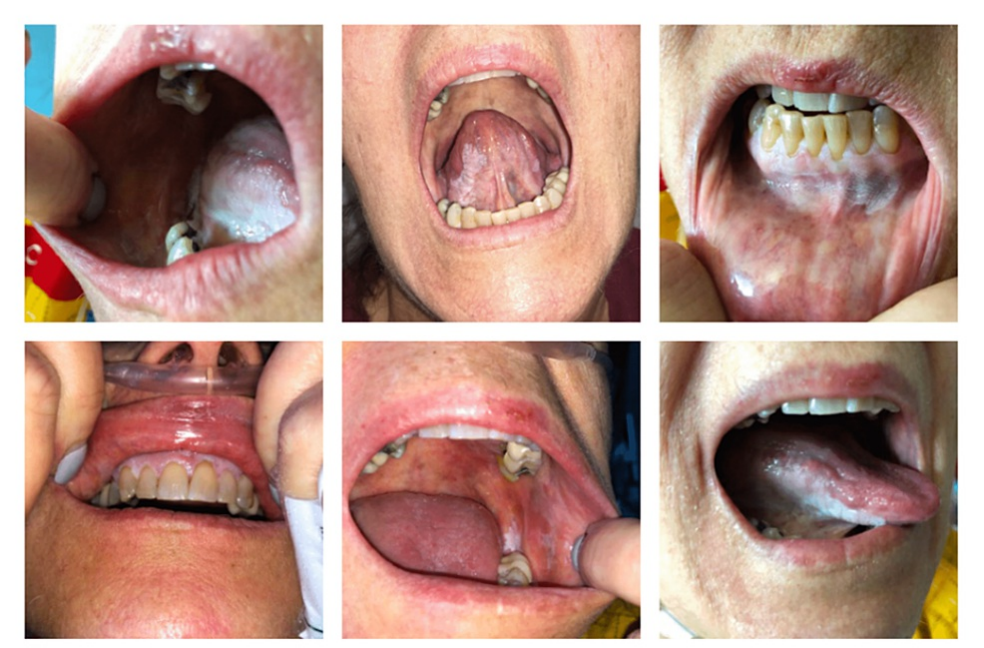
Physical examination of the patient revealed plaque-like lesions with some lace-like patterns focally on the hard palate, buccal mucosa, sublingual region, and gingiva.

On arrival at our institution, her physical examination was significant for plaque-like lesions with some lace-like patterns focally on the hard palate, buccal mucosa, sublingual region, and gingiva (Figure 1). She also had possible involvement in the mucosal aspect of the labia majora. The rest of the physical examination was unremarkable.

**Figure 2:**
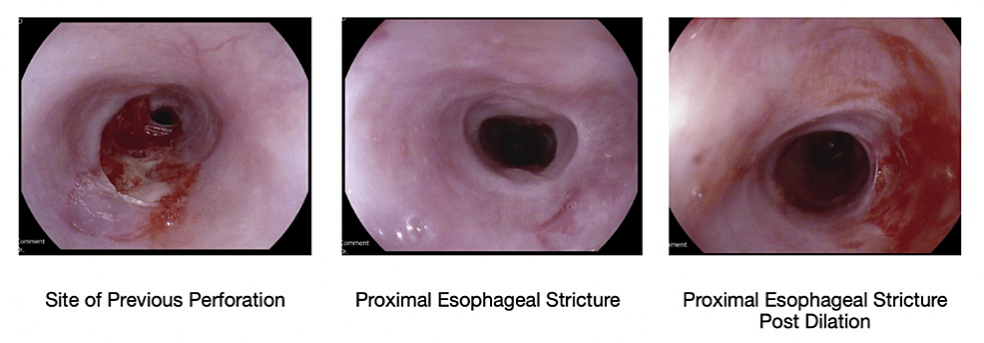
An esophagogastroduodenoscopy revealed edematous mucosa, mucosa peeling, and a thick exudate in the entire examined oropharynx and proximal esophagus. Two severe strictures were found at 18 cm and 22 cm from the incisors.

An esophagram with water-soluble contrast demonstrated a 2 cm long stricture in the upper esophagus without evidence of perforation. A subsequent EGD revealed edematous mucosa, mucosal peeling, and a thick exudate in the entire examined oropharynx and proximal esophagus (Figure 2). Two severe strictures were found at 18 cm and 22 cm from the incisors. The 18 cm stricture was balloon dilated from a diameter of 9-12 mm. The 22 cm stricture measured at a diameter of 7 mm but was not intervened on given an adjacent ulcer with a high risk for perforation. Biopsies were avoided because of the concern for perforation.

The EGD findings were determined to be consistent with ELP. Dermatology was consulted to initiate systemic treatment and obtain further recommendations for management. Punch biopsy of the left buccal mucosa was performed, with results demonstrating lichenoid mucositis without fungal organisms.

**Figure 3:**
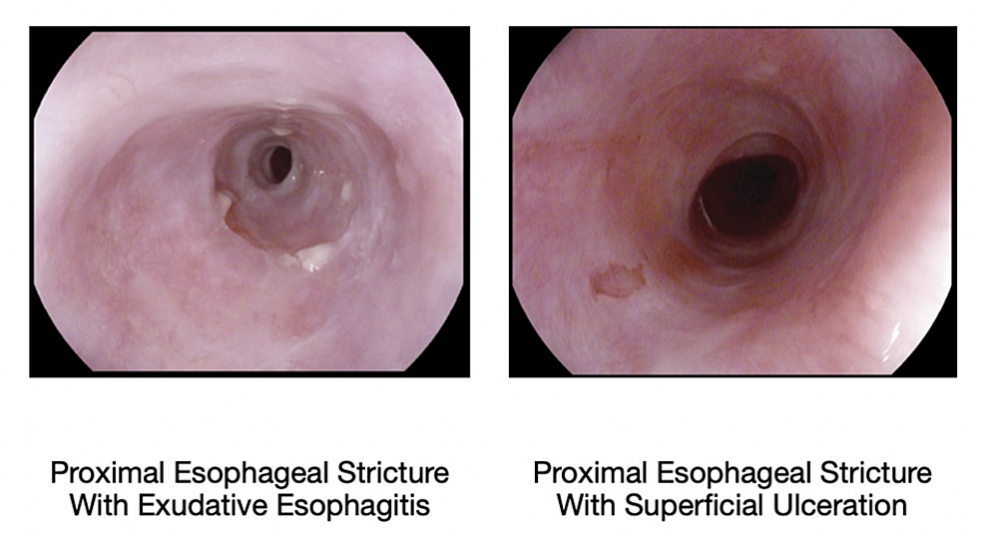
A repeat esophagogastroduodenoscopy (EGD) at four weeks post-discharge for follow-up and dilatation of the strictures revealed improvement of the prior EGD findings.

The patient was treated with oral viscous budesonide swallowed twice daily for the esophageal component and tacrolimus swish and spit for the oral component. Hepatitis B and C serologies and quantiferon gold were obtained in anticipation of starting systemic immunosuppressive or biologic agents as an outpatient. Since NSAIDs and statins are associated with lichen planus, her medications meloxicam and simvastatin were discontinued. The patient improved clinically and tolerated a full liquid diet on discharge. Repeat EGD for follow-up and dilatation of the strictures was done four weeks later. It revealed improvement of the prior findings, and biopsies showed normal squamous mucosa without features of lichen planus (Figure 3). The patient is currently doing well, and is being followed up by both gastrointestinal and dermatology outpatient services.

## Discussion

Esophageal perforation in the setting of ELP is rare. Only one other case identified during the literature review demonstrated perforation, and that was after an attempted dilation [6]. There may be more cases of esophageal perforations that are linked with ELP, but they may be under-diagnosed and attributed to other diseases. In fact, recent studies suggest ELP may be present in up to 50% of patients with lichen planus [7]. Endoscopy is high-risk due to friable mucosa and ulcerations in the esophagus, especially if the underlying disease process is inadequately treated.

Lichen planus is the most common dermatologic condition to involve the esophagus affecting 0.5-2.0% of the general population [4]. Oral lichen planus with esophageal involvement is predominantly a disease of middle-aged Caucasian women [4]. ELP is putatively under-diagnosed and under-reported given that EGDs are not routinely performed in all patients with the disease as mild involvement may not lead to dysphagia. Furthermore, even patients with an EGD may be misdiagnosed as atypical esophagitis, causing delays in treatment [4].

Typical esophageal findings on gross inspection with EGD are a mottled and thickened esophageal mucosa with a white-lacy appearance and loss of the normal vascular pattern [4]. Compared to the linear distal esophagitis that is present in GERD, superficial ulceration with thick, white exudates is present in the proximal esophagus in ELP. The submucosal surface may appear further sloughed and peel away. The presence of these endoscopic findings is highly suggestive of underlying esophageal involvement of lichen planus.

Esophageal strictures are often located in the proximal and mid-esophagus. Most strictures are amenable to dilation with resultant improvement in dysphasia. Medical treatment of lichen planus alone is unlikely to solve pre-existing strictures [4]. Some authors recommend systemic steroids as a first-line treatment given the autoimmune/inflammatory pathophysiology of the disease, and several studies have shown efficacy with high doses [4,8]. Given the deleterious effects of long-term systemic steroid use, studies looking at topical steroids in treating esophageal dysphagia were conducted. Topical steroids such as budesonide and fluticasone have shown a 60% response rate in improving dysphagia [9,10]. There is no unified criteria on the use of systemic steroids versus topical steroids for the treatment of ELP.

Concomitant oral lesions should be evaluated and treated as well. Systemic medications such as antihypertensives, NSAIDs, and oral hypoglycemics have been associated with oral lichenoid lesions [11]. Diagnosis is made clinically, but histological examinations are recommended for confirmation and to exclude dysplasia [12]. Initial practice should include removal of possible offending medications. Treatment of asymptomatic reticular lesions requires observation for progression. Atrophic and ulcerative oral lesions should be treated with topical corticosteroids such as triamcinolone or clobetasol [13]. Calcineurin inhibitors such as tacrolimus and cyclosporine have been used for refractory cases [14]. Treatment is recommended to alleviate accompanying symptoms and diminish the risk of malignant transformation [11].

This patient had known oral lichen planus and esophageal strictures, but these two disease processes were never linked. Therefore, the appropriate treatment was never initiated, eventually leading to a complication of esophageal perforation. It was originally thought the strictures were a complication of GERD and were being treated with a proton pump inhibitor and frequent dilations.

## Conclusions

This is a rare case of esophageal perforation in the setting of ELP. The patient in this case was only diagnosed with ELP and started on appropriate medical therapy after a complication, even though she had undergone many endoscopic evaluations in the past. There must be a high index of suspicion that strictures are a result of atypical disease processes if they are refractory to current medical therapies. ELP will oftentimes be located in the proximal esophagus and have concomitant oral lesions which may aid in diagnosis, such as in this patient. Complications of recurrent esophageal strictures and perforation may be preventable in patients with early diagnosis and adequate treatment.
